# Apigenin Modulates AnxA6- and TNAP-Mediated Osteoblast Mineralization

**DOI:** 10.3390/ijms232113179

**Published:** 2022-10-29

**Authors:** Joanna Mroczek, Slawomir Pikula, Szymon Suski, Lilianna Weremiejczyk, Magdalena Biesaga, Agnieszka Strzelecka-Kiliszek

**Affiliations:** 1 Faculty of Chemistry, University of Warsaw, 1 Pasteur Str., 02-093 Warsaw, Poland; 2 Nencki Institute of Experimental Biology, Polish Academy of Sciences, 3 Pasteur Str., 02-093 Warsaw, Poland

**Keywords:** apigenin, AnxA6, TNAP, mineralization, matrix vesicles, osteoblast, osteosarcoma, atherosclerosis

## Abstract

Mineralization-competent cells like osteoblasts and chondrocytes release matrix vesicles (MVs) which accumulate Ca^2+^ and P_i_, creating an optimal environment for apatite formation. The mineralization process requires the involvement of proteins, such as annexins (Anx) and tissue-nonspecific alkaline phosphatase (TNAP), as well as low molecular-weight compounds. Apigenin, a flavonoid compound, has been reported to affect bone metabolism, but there are doubts about its mechanism of action under physiological and pathological conditions. In this report, apigenin potency to modulate annexin A6 (AnxA6)- and TNAP-mediated osteoblast mineralization was explored using three cell lines: human fetal osteoblastic hFOB 1.19, human osteosarcoma Saos-2, and human coronary artery smooth muscle cells HCASMC. We compared the mineralization competence, the morphology and composition of minerals, and the protein distribution in control and apigenin-treated cells and vesicles. The mineralization ability was monitored by AR-S/CPC analysis, and TNAP activity was determined by ELISA assay. Apigenin affected the mineral structure and modulated TNAP activity depending on the concentration. We also observed increased mineralization in Saos-2 cells. Based on TEM-EDX, we found that apigenin influenced the mineral composition. This flavonoid also disturbed the intracellular distribution of AnxA6 and TNAP, especially blocking AnxA6 aggregation and TNAP attachment to the membrane, as examined by FM analysis of cells and TEM-gold analysis of vesicles. In summary, apigenin modulates the mineralization process by regulating AnxA6 and TNAP, as well as through various effects on normal and cancer bone tissues or atherosclerotic soft tissue.

## 1. Introduction

Mineralization-competent cells, that is, osteoblasts in the bone and chondrocytes in the cartilage, participate in the initial steps of mineral formation during intramembranous and endochondral ossification, respectively [1]. These mineral-forming cells produce extracellular matrix (ECM) proteins and release matrix vesicles (MVs), which initiate mineralization [2,3]. MVs, having both a similar lipid composition and protein profile to parent cells [4,5,6,7] and containing relatively high concentrations of Ca^2+^ and inorganic phosphate (P_i_), create an optimal environment to induce the formation of hydroxyapatite (HA) [8,9,10,11]. The minerals are subsequently released into the ECM, where their further growth takes place [3,12].

The mineralization process requires the involvement of various proteins, such as annexins (Anx) [6,13] and tissue-nonspecific alkaline phosphatase (TNAP). TNAP is an enzyme that can be found in a soluble form or anchored to the plasma membranes through a glycosylphosphatidylinositol (GPI) anchor, which provides membrane mobility and facilitates the release of TNAP by phospholipases [14,15,16]. Its huge role in mineralization is based on its ability to hydrolyze inorganic pyrophosphate (PP_i_), a strong inhibitor of apatite formation, into P_i_ [8,17]. Together with ectonucleotide pyrophosphatase/phosphodiesterase 1 (NPP1) hydrolyzing ATP to PP_i_, TNAP is the main regulator of the PP_i_/P_i_ homeostasis [8,18]. Annexins are proteins which bind calcium ions and phospholipids, and some of them have been reported to be present in MVs [4,19,20,21,22]. They are found in the MV lumen or bound to the internal or external surface of the phosphatidylserine (PS)-rich membrane of MVs [6,8]. Annexin A6 (AnxA6), the largest of the annexins and the only one composed of eight domains instead of four [23,24], may also be inserted in the membrane bilayer [23] and, under acidic pH, can form ion channels across the membrane permeable to calcium ions [8,22]. An important role is also played by low molecular-weight (LMW) compounds, which can act as activators or inhibitors of mineral formation.

Flavonoids are organic compounds found in foods of plant origin [25]. They have a beneficial effect on the human body, counteracting oxidative stress through their ability to scavenge free radicals [26,27,28]. Flavonoids also have anti-inflammatory, anti-tumor, and neuroprotective activities [29,30,31]. Several flavonoids have also been reported to affect processes regulating bone metabolism. They have the ability to modulate enzyme activity [32,33,34,35,36,37,38], interact with ECM proteins and cell-surface receptors [39], and to interact with transcription factors resulting in changes in gene expression [40]. Apigenin (4′,5,7-trihydroxyflavone) is a flavonoid belonging to the flavone class present in fruits and vegetables, such as onion, parsley, oranges, tea, or wheat sprouts [41,42,43,44]. In recent years, apigenin has gained a great interest because of its differential effects on normal and cancer cells when compared with other flavonoids [41,45]. Various studies have provided evidence that apigenin possesses antioxidant, anti-mutagenic, anti-carcinogenic, anti-inflammatory and anti-proliferative properties [41,46]. There have also been reports of the effect of apigenin in human mesenchymal stem cells [32,33] and osteoblastic MC3T3-E1 cells from ovariectomized mice [34] or other flavonoids: quercetin in ROB (rat calvarial osteoblast-like) cells [35], myricetin in hFOB cells and human osteosarcoma MG-63 cell line [36], epigallocatechin-3-gallate in Saos-2 cells [37], and hesperetin in primary rat osteoblasts [38] on bone metabolism. Zhang et al. [32] observed that apigenin promotes osteogenic differentiation of mesenchymal stem cells (hMSCs) and regulates signaling pathways involving JNK and p38 MAPK. Studies also showed that apigenin affects TNAP activity and the amount of minerals produced. This is also consistent with research carried out by Liao et al. [47] on mouse macrophage ANA-1 cells. Jung et al. [48] observed a protective effect of apigenin against oxidative stress-induced damage in osteoblastic cells. This effect was due to its antioxidant activity and ability to enhance differentiation by increasing the expression of osteoblastic differentiation genes such as TNAP, collagen, osteopontin, osteoprotegerin, bone sialoprotein, osterix, and osteocalcin and bone morphogenetic proteins genes such as BMP2, BMP4, and BMP7 in mice osteoblasts MC3T3-E1. On the other hand, Goto et al. [34] reported a decrease in collagen production, TNAP activity, and the amount of calcium deposits after incubation of MC3T3-E1 cells with apigenin and concluded that apigenin inhibited osteoblastogenesis and osteoclastogenesis in ovariectomized mice. Studies of the effects of apigenin on the mineralization process are of increasing interest to scientists; however, the mechanism of action of this flavonoid under physiological and pathological conditions has not been elucidated so far.

In the present study, we have examined the effect of apigenin on AnxA6- and TNAP-mediated osteoblast mineralization using three cellular models: human fetal osteoblastic hFOB 1.19, human osteosarcoma Saos-2, and human coronary artery smooth muscle cells HCASMC. We investigated the apigenin action on the mineralization process occurring in MVs through the regulation of AnxA6 and TNAP, that is, two proteins crucial in this process. To explore the possible mechanisms, we compared the mineralization competence of cells, their morphology, the composition of minerals, and protein distribution in cells and vesicles.

## 2. Results

### 2.1. Characterization of the Morphology of Human Fetal Osteoblastic Cell Line (hFOB 1.19 Cells) and Osteosarcoma Cell Line (Saos-2 Cells)

**Figure 1:**
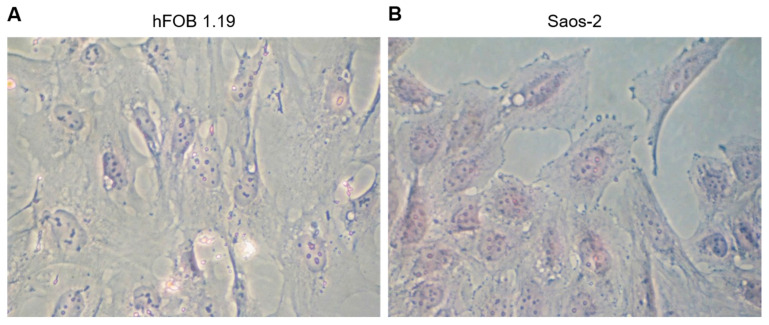
Morphology of hFOB 1.19 (**A**) and Saos-2 (**B**) cells. Hematoxylin–eosin staining of cells fixed in paraformaldehyde. Cells were observed under an optical microscope (magnification 400×).

Human fetal osteoblastic hFOB 1.19 and osteosarcoma Saos-2 cells are good models of bone physiological and pathological mineralization, respectively, compared to human coronary artery smooth muscle cells (HCASMC) which are a good model of pathological mineralization of soft tissues. Both cell lines are competent in the mineral formation process and are widely used in in vitro studies [10,49]. hFOB 1.19 cells are bone osteoblasts isolated from fetus limb and transfected with SV40 large T antigen. Saos-2 cells are human osteoblast-like cells isolated from bone tumors in osteosarcoma. Morphological analysis of tested cells was performed by hematoxylin–eosin staining and observation under a light microscope (Figure 1). hFOB 1.19 cells (Figure 1A) were elongated with sharply ended appendages and a peripherally located nucleus, while Saos-2 cells (Figure 1B) had a rhombic shape, rounded tabs, and a centrally located nucleus.

### 2.2. Effect of Apigenin on hFOB 1.19, Saos-2 and HCASMC Cell Proliferation

**Figure 2:**
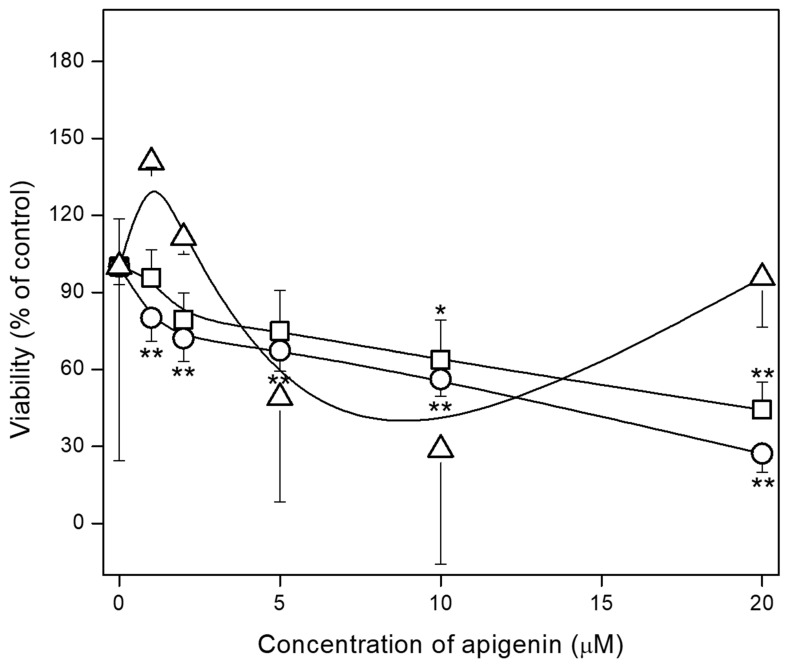
The effect of apigenin on viability of hFOB 1.19 (squares), Saos-2 (circles) and HCAMSC (triangles) cells. The cells were incubated with different concentrations (µM) of apigenin for 7 days (hFOB 1.19 and Saos-2 cells) or 14 days (HCAMSC cells), then MTT assay was performed. Data are means ± S.E. of at least three independent experiments (* *p* < 0.05, ** *p* < 0.01 compared with cells without apigenin).

Cells were incubated in culture medium with different concentrations of apigenin (1–20 µM) for 1, 3, 5, 7, or 14 days. Dose–response and time–course analysis were performed to determine the effect of apigenin on cell proliferation using MTT assay [50]. The treatment of both types of cells with apigenin for 7–14 days decreased the number of viable cells in a dose-dependent manner with stronger effects on Saos-2 cells (Figure 2). The differences in hFOB 1.19 (Figure 2, squares) and Saos-2 (Figure 2, circles) cell numbers were significant from the 10 and 1 µM apigenin dose, respectively. For HCASMC, differences in the cell number were visible only after 5 and 10 µM apigenin doses (Figure 2, triangles). The IC50 value of apigenin determined from the curve for hFOB 1.19 cells was 17.1 µM and for Saos-2 cells was 11.7 µM, respectively [51].Analysis of the apigenin effect at concentrations of 0, 1, 2, 5, 10, and 20 µM on both cell cultures over time (Table S1A,B) confirmed that control osteoblastic hFOB 1.19 and osteosarcoma Saos-2 continue to proliferate rapidly during the 14 days of culture, with a higher growth rate of Saos-2 cells, while the proliferation of cells treated with 1, 2, 5, and 10 µM apigenin is slowed. An apigenin concentration of 20 µM caused changes to cell morphology, inhibition of proliferation, and cell death of both types of cells and was not used in subsequent experiments. The effect of apigenin on HCASMC culture over time (Table S1C) showed that control atherosclerotic cells also continue to proliferate rapidly during the 21 days of culture, while the proliferation of cells treated with 5 and 10 µM apigenin is slowed.

### 2.3. Effect of Apigenin on Mineralization and TNAP Activity of hFOB 1.19 and Saos-2 Cells

**Figure 3:**
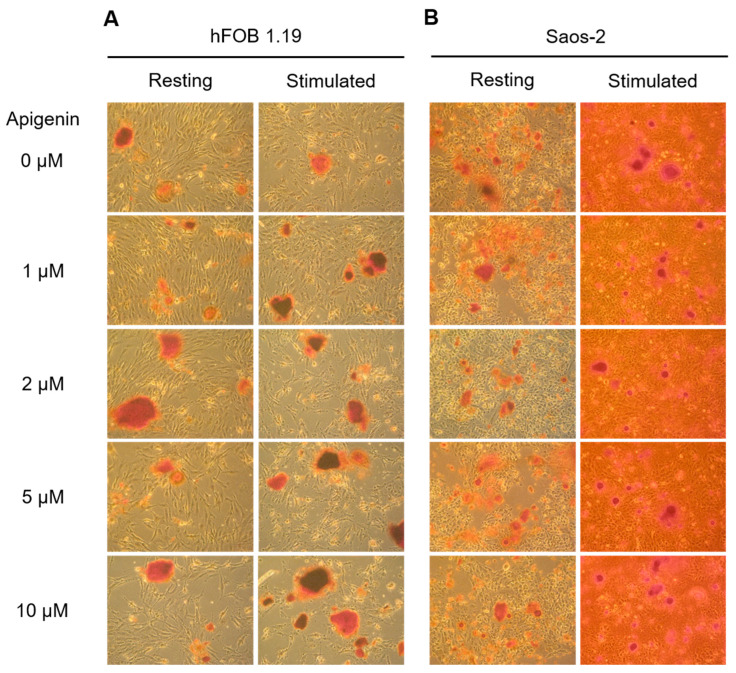
The effect of apigenin on mineralization of hFOB 1.19 (**A**) and Saos-2 (**B**) cells under resting conditions or in the presence of stimulators, AA and β-GP. Cells were incubated with different concentrations (µM) of apigenin for 7 days and then stained with AR-S and observed under an optical microscope (magnification 100×).

**Figure 4:**
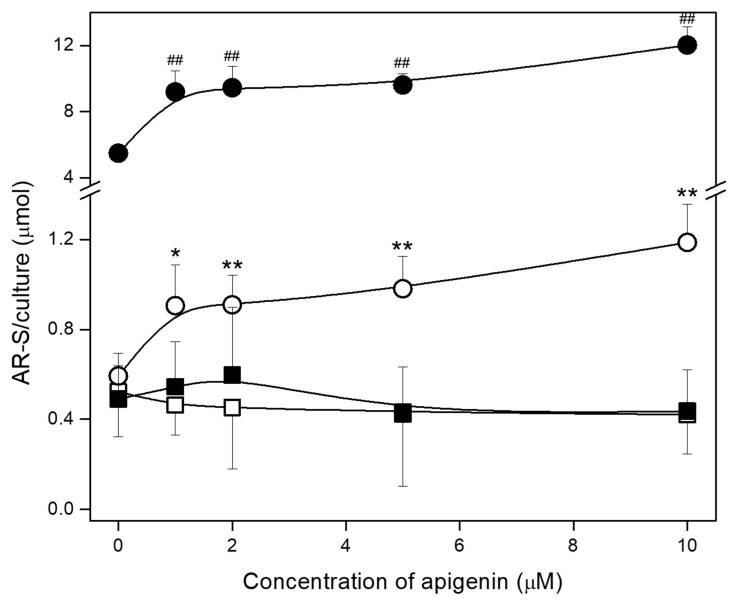
The effect of apigenin on the mineralization of hFOB 1.19 and Saos-2 cells. Quantitative analysis of calcium deposits in hFOB 1.19 (squares) and Saos-2 (circles) cells under resting conditions (open squares/circles) or in the presence of stimulators, AA, and β-GP (filled squares/circles) was carried out by staining with AR-S, de-staining with CPC, and absorbance measurements at λ 562 nm. The degree of mineralization was normalized to the relative number of viable cells. Data are means ± S.E. of at least three independent experiments (* *p* < 0.05, **/## *p* < 0.01 compared with resting/stimulated cells without apigenin).

Cells were cultured for 7 days without stimulators of mineralization (Resting, R) or after stimulation with 50 µg/mL ascorbic acid (AA) and 7.5 mM β-glycerophosphate (β-GP) (Stimulated, S). The mineralization process was also modulated by the addition of different concentrations of apigenin to resting and stimulated cells starting 4 h after addition of AA and β-GP. The ability of cells to mineralize was confirmed by alizarin red S/cetylpyridinium chloride (AR-S/CPC) analysis and determination of TNAP activity by ELISA assay. Differences in calcium deposit formation were observed by AR-S staining under an optical microscope (Figure 3). Apigenin affected the mineral structure, making it more compact. Minerals produced by hFOB 1.19 cells (Figure 3A) also had darker centers after the addition of apigenin, especially under stimulating conditions. Saos-2 cells (Figure 3B) showed significantly increased mineralization in stimulated cultures (Figure 3B, right panel) in comparison to resting cultures (Figure 3B, left panel) even without apigenin. Minerals formed by osteosarcoma cells (Figure 3B) were also more rounded than those produced by osteoblasts (Figure 3A), had dark centers in all cases, and spread elevated areola around the centers with increasing apigenin concentrations. Quantitative analysis of calcium nodules by CPC de-staining confirmed that Saos-2 cells (Figure 4, circles) had a stronger ability to mineralize than hFOB 1.19 cells (Figure 4, squares), and their stimulation (Figure 4, filled circles/squares) caused almost ten times more effective mineralization in comparison to resting cells (Figure 4, open circles/squares). In turn, the addition of apigenin in all of the tested concentrations increased the amount of minerals in both resting and stimulated Saos-2 cultures (Figure 4, open and filled circles), while it had no effect on hFOB 1.19 cultures (Figure 4, open and filled squares).

**Figure 5:**
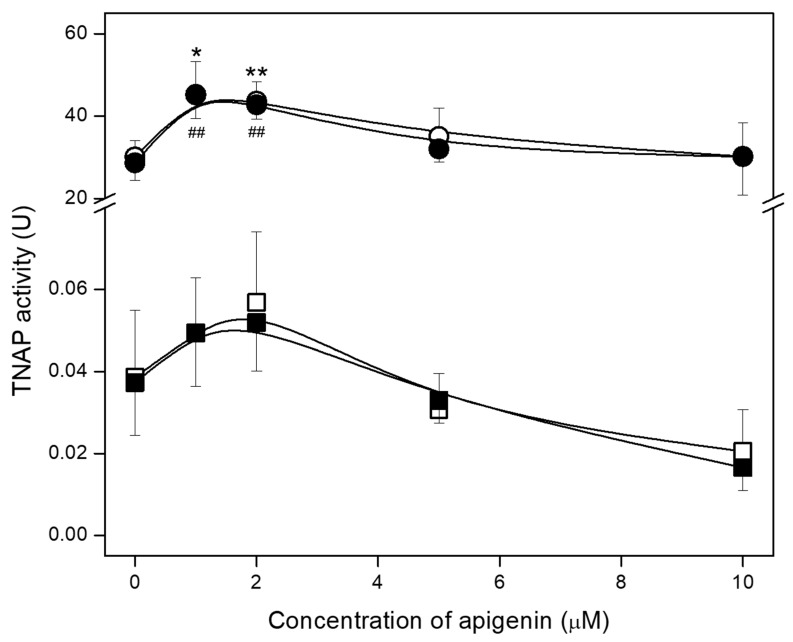
The effect of apigenin on the TNAP activity of hFOB 1.19 (squares) and Saos-2 (circles) cells under resting conditions (open squares/circles) or in the presence of stimulators, AA, and β-GP (filled squares/circles). Cells were incubated with different concentrations (µM) of apigenin, and the TNAP activity assay (ALP Yellow pNPP Liquid Substrate System for ELISA) was performed in whole cell lysates after 7 days of culture. Absorbance at λ 405 nm was measured for 60 min with 15 s intervals at 37 °C. The TNAP activity values were normalized to the relative number of viable cells. Data are means ± S.E. of at least three independent experiments (* *p* < 0.05, **/## *p* < 0.01 compared with resting/stimulated cells without apigenin).

The ability to mineralize was also confirmed by determination of TNAP activity in whole cell lysates. In all cases TNAP activity in osteosarcoma Saos-2 was much higher (almost 10^3^ times) than in osteoblastic hFOB 1.19 cells (Figure 5, circles and squares) [10,13]. In both cell lines, there were no significant differences between resting and stimulated cells (Figure 5, open and filled circles/squares). Treatment with low apigenin concentrations, that is 1 and 2 µM, slightly increased TNAP activity in both types of cells cultured under resting and stimulated conditions when compared with control cells (Figure 5, circles/squares). Higher concentrations of apigenin had no effect on the activity of TNAP in Saos-2 cells (Figure 5, circles), while they slightly decreased it in both resting and stimulated hFOB 1.19 cells (Figure 5, open and filled squares).

### 2.4. Effect of Apigenin on Protein Profiles of hFOB 1.19 and Saos-2 Cells

Lysates of 5 *×* 10^8^ cells per each experimental variant were homogenized in Triton Lysis Buffer (TLB) (as described in Section 4.5) and centrifuged. The pellets were analyzed by WB in order to determine the protein content (Figure S1A). We observed an increase in AnxA6 (70 kDa) and TNAP (around 70 kDa) relative to actin (39 kDa) level after stimulation of hFOB 1.19 and Saos-2 cells. The level of TNAP was a little higher in Saos-2 cells than in hFOB 1.19 cells (Figure S1B, open bars), whereas the level of AnxA6 was almost two times higher in Saos-2 than in hFOB 1.19 cells (Figure S1B, grey bars). Moreover, TNAP content remained at a low level (below1.0) in resting and stimulated hFOB 1.19 cells, whereas its level was intermediate (around 1.0) in both resting and stimulated Saos-2 cells (Figure S1B, open bars). AnxA6 remained at a medium level (around 1.0) in resting hFOB 1.19 cells, whereas its level was higher (above 1.0) in stimulated hFOB cells as well as in both resting and stimulated Saos-2 cells (Figure S1B, grey bars). After the addition of apigenin, we did not observe any statistically significant changes in the profiles of the examined proteins as compared with control cells without flavonoid (Figure S1B). Only the highest tested concentration (10 µM) of apigenin slightly influenced the content of both proteins, decreasing AnxA6 level in stimulated hFOB 1.19 cells and increasing TNAP level in stimulated Saos-2 cells.

### 2.5. Effect of Apigenin on Protein Distribution in hFOB 1.19 and Saos-2 Cells

**Figure 6:**
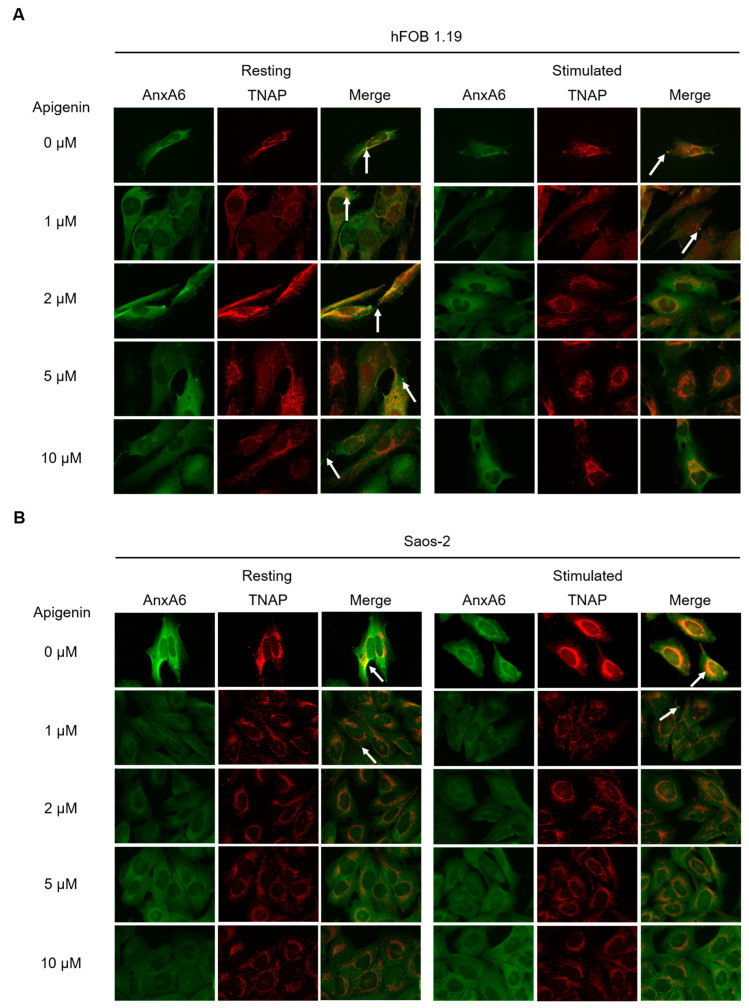
Co-localization of AnxA6 and TNAP during mineralization of hFOB 1.19 (**A**) and Saos-2 (**B**) cells under resting conditions or in the presence of stimulators, AA and β-GP. Cells were incubated with different concentrations (µM) of apigenin for 7 days, fixed and analyzed by fluorescent microscopy (magnification 630×). AnxA6 (green) was immunostained with anti-AnxA6 primary antibody conjugated with Alexa Fluor 488 secondary antibody. TNAP (red) was immunostained with anti-TNAP primary antibody conjugated with Alexa Fluor 594 secondary antibody. Sites of AnxA6 and TNAP co-localization are visible in yellow on merge images (arrows).

In order to determine the influence of apigenin on the distribution of selected proteins engaged in the calcification process, immunocytochemical staining was performed. Immunochemistry analysis specific to AnxA6 (green: Alexa Fluor 488) and TNAP (red: Alexa Fluor 594) was carried out using appropriate primary antibodies followed by fluorophore-conjugated secondary antibodies. In control variants without apigenin, in resting osteoblastic hFOB 1.19 (Figure 6A, upper left side), and osteosarcoma Saos-2 (Figure 6B, upper left side) cells, AnxA6 was uniformly distributed, as reported earlier [13]. After stimulation to mineralization, AnxA6 in hFOB 1.19 cells (Figure 6A, upper right side) aggregated in the cytoplasm, while in stimulated Saos-2 cells (Figure 6B, upper right side), it translocated to the inner surface of the cellular membrane [11,13]. In resting hFOB 1.19 cells (Figure 6A, upper left side), TNAP was localized in the perinuclear region, whereas in resting Saos-2 cells (Figure 6B, upper left side) along the membrane, where it co-localized with AnxA6 (Figure 6B, upper left side, yellow color, arrow). After stimulation, a fraction of TNAP in hFOB 1.19 cells (Figure 6A, upper right side, yellow color, arrow) and Saos-2 cells (Figure 6B, upper right side, yellow color, arrow) co-localized with aggregates of AnxA6 [13]. The addition of apigenin affected cell morphologies, making cells more rounded, especially in the case of stimulated hFOB 1.19 cells (Figure 6A, right side) and both resting and stimulated Saos-2 cells (Figure 6B). In resting hFOB 1.19 cells, addition of apigenin slightly affected the distribution of the tested proteins, resulting in a more uniform TNAP localization and higher co-localization with AnxA6 in the presence of low apigenin concentration within the 1–2 µM range (Figure 6A, left side, yellow colors, arrows). In stimulated hFOB 1.19 cells in the presence of 1 µM of apigenin, there were no significant differences compared to cells without apigenin treatment, while higher concentrations of this flavonoid affected the intracellular distribution of AnxA6 and TNAP, causing their lower aggregation and co-localization (Figure 6A, right panel). Alterations in protein distribution for both resting and stimulated Saos-2 cells in the presence of apigenin (Figure 6B) occurred already at the concentration of 1 µM, and higher concentrations increased this effect. The addition of this flavonoid reduced AnxA6 aggregation and blocked TNAP attachment to the membrane. Relative co-localization areas, calculated from FM images, showed that, for both cell lines without addition of apigenin, stimulation increased AnxA6 and TNAP co-localization (Table 1) [13]. Values obtained for Saos-2 cells, for both resting and stimulated variants, were higher, than for hFOB 1.19 cells (Table 1). Treatment with apigenin at all tested concentrations sharply decreased the relative co-localization areas between AnxA6 and TNAP in Saos-2 cells, both resting and stimulated. The addition of 1 and 2 µM of apigenin to hFOB 1.19 resting cells slightly increased the co-localization of tested proteins, while higher concentrations of this flavonoid, that is 5 and 10 µM, had no significant effect. The stimulated hFOB 1.19 cells were indifferent to the action of 1 and 2 µM of apigenin, while in the presence of 5 and 10 µM of apigenin, the relative co-localization areas were decreased when compared with control stimulated cells (Table 1).

### 2.6. Effect of Apigenin on Vesicles and Minerals Produced by hFOB 1.19 and Saos-2 Cells

**Figure 7:**
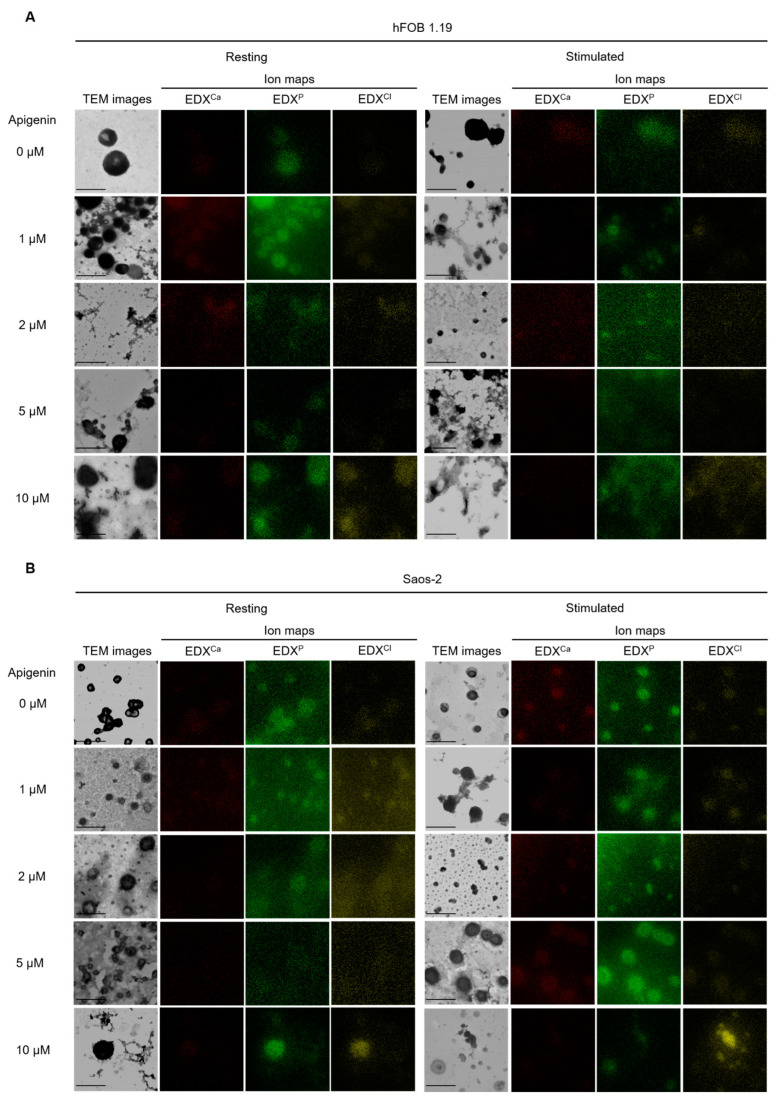
TEM images and MV chemical composition during the mineralization of hFOB 1.19 (**A**) and Saos-2 (**B**) cells under resting conditions or in the presence of stimulators, AA and β-GP. Cells were incubated with different concentrations (µM) of apigenin for 7 days, lysed, and analyzed by TEM-EDX (magnification 50,000×). Ion maps for Ca (red), *p* (green), and Cl (yellow) from EDX. Bar: 1 μm.

Cells were cultured without stimulators of mineralization (resting) or after stimulation with 50 µg/mL ascorbic acid (AA) and 7.5 mM β-glycerophosphate (β-GP) (stimulated) and treated with different concentrations of apigenin for 7 days. The effect of apigenin on the vesicles (all microparticles including MVs) and minerals produced by both cell lines was determined by TEM-EDX analysis (Figure 7). TEM images indicated the presence and number of vesicles in hFOB 1.19 (Figure 7A) and Saos-2 (Figure 7B) cells, while EDX ion maps showed calcium (red points), phosphorus (green points), and chlorine (yellow points) distributions (Figure 7). In control variants without apigenin, TEM images showed that Saos-2 cells (Figure 7B) produced more small- or medium-sized vesicles (diameter < 500 nm) than hFOB 1.19 cells (Figure 7A), in which large-sized vesicles (diameter > 500 nm) were more frequently observed. Saos-2 cells (Figure 7B) also had a higher calcium and phosphorus content in vesicles compared with hFOB 1.19 cells (Figure 7A), especially under stimulated conditions (Figure 7B, right panel) [52]. The addition of apigenin to hFOB 1.19 cells (Figure 7A) caused vesicle aggregation, especially in the case of resting cells (Figure 7A, left panel). Similarly, TEM images showed vesicle chains in resting Saos-2 cells, mainly after treatment with 5 µM of apigenin (Figure 7B, left panel). Mapping of calcium, phosphorus, and chlorine by EDX indicated that apigenin slightly affected the composition of minerals in hFOB 1.19 cells (Figure 7A), causing an increase in the chlorine content, mainly at the apigenin concentration of 10 µM, while in Saos-2 cells (Figure 7B) already caused a significant increase in the presence of 1 µM apigenin.

### 2.7. Effect of Apigenin on Protein Distributions in Vesicles Released by hFOB 1.19, Saos-2 and HCASMC Cells

**Figure 8:**
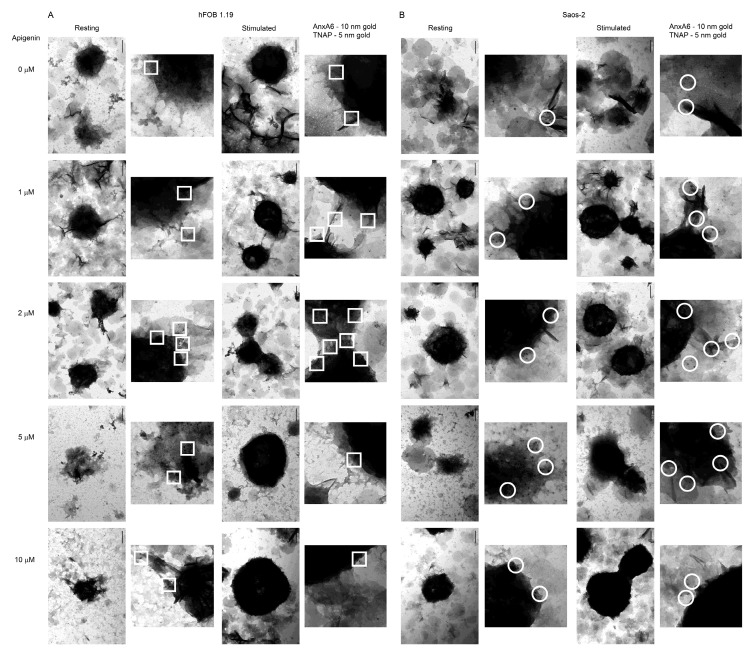
TEM images of the co-localization of AnxA6 and TNAP in MVs during the mineralization of hFOB 1.19 (**A**) and Saos-2 (**B**) cells under resting conditions or in the presence of stimulators, AA and β-GP. Cells were incubated with different concentrations (µM) of apigenin for 7 days, lysed, and analyzed by TEM (magnification 100,000×). Bar: 200 nm. Additional magnifications 300,000×. AnxA6 was labelled with anti-AnxA6 primary antibody conjugated with 10 nm colloidal gold secondary antibody. TNAP was labelled with anti-TNAP primary antibody conjugated with 5 nm colloidal gold secondary antibody. Sites of AnxA6 and TNAP co-localization are marked by squares for hFOB 1.19 cells and circles for Saos-2 cells.

In order to confirm the influence of apigenin on the distribution of selected proteins in vesicles, immunogold labelling was performed. TEM-gold analysis specific to AnxA6 (5 nm particles) and TNAP (10 nm particles) was carried out using the appropriate primary antibodies followed by colloidal gold-conjugated secondary antibodies. Obtained images allowed us to explore the distribution and co-localization of proteins inside and around the vesicles (Figure 8). Results were in agreement with those obtained for cells. For both cell lines stimulation increased AnxA6 and TNAP co-localization in comparison with resting cells. In the presence of low concentrations of apigenin (1 and 2 µM), the tested proteins co-localize within vesicles, especially in those released by hFOB 1.19 cells (Figure 8A, squares). In the case of higher concentrations (5 and 10 µM), there was a small amount of co-localized proteins on the surface and around vesicles, which indicated that most of them were probably locked inside vesicles (Figure 8A,B, squares and circles). The increase in apigenin concentration also caused vesicle aggregation and the retention of minerals inside vesicular structures in both cell types. Vesicles were highly mineralized and did not break (Figure 8A,B). For the HCASMC cell line, stimulation also increased AnxA6 and TNAP co-localization when compared with resting cells (Figure S2). Only higher concentrations (5 and 10 µM) of apigenin were tested, and a small number of co-localized proteins on the surface and around vesicles was also visible, indicating their locking inside vesicles (Figure S2, triangles).

## 3. Discussion

This study describes the apigenin effect on the proliferation as well as AnxA6- and TNAP-mediated mineralization of human fetal osteoblastic hFOB 1.19 and osteosarcoma Saos-2 cells. Elongated hFOB 1.19 cells with peripherally located nuclei and rhombic Saos-2 cells with centrally located nuclei (Figure 1) provide good models of physiological and pathological mineralization, respectively, as was previously reported [5,6,10,11,13].

Time–course analysis of cell viability after incubation with apigenin demonstrated that hFOB 1.19 and Saos-2 cells proliferated rapidly during the 14 days of culture, while treatment with apigenin (1–20 µM) attenuated cell growth, and the 20 µM concentration changed the morphology of cells and inhibited proliferation (Table S1). After 7 days of incubation with apigenin, the viability of osteoblastic hFOB 1.19 and osteosarcoma Saos-2 cells was decreased in a dose-dependent manner with a stronger effect on Saos-2 cells (Figure 2). These results confirm the anti-tumor properties of apigenin and its ability to induce apoptosis as well as its various effects on healthy and cancer cells [41].

We compared the mineralization ability of both types of the studied cells. AR-S staining of calcium deposits revealed the effect of apigenin on the mineral structure, making it more compact (Figure 3). Nodules produced by hFOB 1.19 cells (Figure 3A) also had darker centers after incubation of apigenin, while these formed by Saos-2 cells (Figure 3B), spread elevated areola around the centers with increasing apigenin concentrations. Stimulated Saos-2 cells showed 10 times higher rate of mineralization than stimulated hFOB 1.19 cells, probably correlated with almost 1000-times higher TNAP activity, in agreement with previous studies [5,6,10,13]. The addition of apigenin increased the amount of minerals in both resting and stimulated Saos-2 cells (Figure 4, open and filled circles) with the greatest leap noted after adding 1 µM of apigenin and followed by a flatter increase at higher concentrations. On the other hand, apigenin had no significant effect on the mineralization of hFOB 1.19 cells (Figure 4, open and filled squares). These results also correspond to an increase in TNAP activity in the presence of 1 and 2 µM apigenin (Figure 5). In Figure 4 (AR-S staining of minerals) and Figure 5 (TNAP activity test), some deviations are present only for hFOB 1.19 cells and mainly after 2 μM apigenin dose because of differences in cell densities which is are presented in Supplementary Materials as Table S1 (MTT viability test) for control variant without apigenin treatment. Our findings confirmed that TNAP and hydrolysis of PP_i_ into P_i_ plays a significant role in the mineralization process, and that modulation of activity of this enzyme may result in changes to the amount of formed minerals [10,13,32]. What is more, an increase in TNAP activity accompanied by a decrease in proliferation may suggest that apigenin treatment promotes cell differentiation into more mature bone-forming cells [53,54]. Furthermore, addition of apigenin caused upregulation of osteoblast differentiation genes, including TNAP, OPN, OPG, BSP, OSX, OC, and BMPs, in mice MC3T3-E1 osteoblastic cells. BMPs are multifunctional growth factors, which, among other things, modulate ALP activity, the synthesis of collagen and OC, and the stimulation of their production by apigenin suggests its function in cell differentiation [48].

The stimulation of cells for mineralization enhanced the expression level of AnxA6, mainly in hFOB 1.19 osteoblasts, and of TNAP, mainly in Saos-2 cells, which is consistent with the increased mineralization ability of both these cell lines upon stimulation. AnxA6 level increased in Saos-2 cells almost two times compared to hFOB 1.19 cells. Treatment of both cell types with apigenin slightly increased TNAP level in Saos-2 cells, whereas AnxA6 level decreased in hFOB 1.19 cells upon the addition of this flavonoid (Figure S1A,B).

The stimulation of mineralization increased AnxA6 aggregation (Figure 6A,B, upper right sides) and its co-localization with TNAP, mainly in Saos-2 cells (Table 1) showing strong mineralization, which is consistent with previous reports [11,13]. Treatment of resting hFOB 1.19 cells with apigenin only slightly affected distribution of the tested proteins (Figure 6A, left side) and increased their co-localization in the presence of 1 and 2 µM flavonoid (Table 1). In stimulated hFOB 1.19 cells there were also no significant differences after the addition of apigenin, which only caused a lower degree of co-localization of AnxA6 and TNAP in higher concentrations (Table 1). This is consistent with the lack of a significant effect of apigenin on mineralization ability and TNAP activity in hFOB cells (Figure 4 and Figure 5). Treatment of Saos-2 cells with apigenin, both in resting and stimulated conditions, blocked AnxA6 aggregation and TNAP attachment to the membrane (Figure 6B), also resulting in low co-localization of these proteins, even in the lowest tested concentration (Table 1).

Recent data based on X-ray microanalysis and IR spectroscopy demonstrated significant differences between minerals formed by hFOB 1.19 and Saos-2 cells. It was found that hFOB 1.19 cells produce amorphous calcium phosphate complexes, while Saos-2 cells produce mixtures of amorphous calcium phosphate complexes and apatites with a Ca/P ratio close to the theoretical value for HA [10,13]. The results obtained in this report also suggest that apigenin probably alters the composition of calcium deposits, especially in the case of Saos-2 cells, causing the formation of some non-specific minerals. We already identified a strong increase in chlorine content after the addition 1 µM apigenin to Saos-2 culture (Figure 7B).

Immunogold labelling performed in order to explore AnxA6 and TNAP distribution in vesicles confirmed results obtained for cells. Treatment with 1 or 2 µM apigenin caused no significant differences and the tested proteins co-localized within vesicles, while higher concentrations induced a decrease in the amount of TNAP on the vesicle surface. TEM images pointed to vesicle aggregation and retention of the minerals in bone and soft tissues with increasing apigenin concentrations (Figure 8A,B and Figure S2). Our findings suggest that despite enhanced mineralization induced by apigenin, the minerals cannot be released to ECM.

We hypothesize that the probable mechanism of action of flavonoids is carried out in the regulation of reactive oxygen species (ROS) and immunomodulation. There are numerous studies that underline their influence on the nuclear factor kappa–light-chain-enhancer of activated B cells (NF-κB), mitogen-activated protein kinase (MAPK) and arachidonic acids and phosphatidylinositide 3-kinase B (PI3K/AKT) as an inhibitor. On the other hand they regulate superoxide dismutase (SOD), catalase and glutathione peroxidase (GPx) expression and nuclear factor erythroid related factor 2 (Nrf2) [55]. The authors recommend the use of fruit and vegetables in the diet and to consider the intake ranging from 50 to 200 mg/day of polyphenols (naringenin, apigenin, kaempferol, hesperidin, ellagic acid, and oleuropein), an amount that can also be doubled in pathological conditions to ensure a beneficial action, at least from an anti-inflammatory and antioxidant viewpoint [55]. The presence of microRNA (miRNAs) in MVs suggests that MVs can function as signalosomes in cell–cell communication during cartilage and bone development via transfer of specific miRNAs. However, it remains to be determined whether such cell–cell communication occurs in vivo [8].

The mechanism of apigenin action on the mineralization process through annexins and TNAP signaling pathways seems to be complicated and limits this work. We demonstrated earlier by immunofluorescence the presence of membrane-bound TNAP that seems to form clusters on the plasma membrane of vascular smooth muscle cells (VSMCs) from rat MOVAS cell line cultured in mineralizing conditions. Our results suggested a role for collagen and cholesterol-enriched lipid rafts then AnxA2 and AnxA6 in promoting calcification induced by TNAP in atherosclerotic plaques [56]. Data showing the effect of apigenin on annexins and TNAP genes and proteins’ expressions are missing and are planned to be analyzed in the near future. The results we presented were obtained in vitro, and it is necessary to perform further experiments in vivo to improve the data proposed in this paper and to find the concentrations of apigenin possible to use during future therapies of pathological mineralization. We focused on in vitro experiments, which are the first step in looking for an explanation of the mechanisms of the mineralization process, and which have some limitations for drug targeting. Translation of the presented results to an in vivo environment rich in apigenin requires more experiments in order to confirm its potential as a drug and to find resolutions for delivering this drug to its destinations.

**Figure 9:**
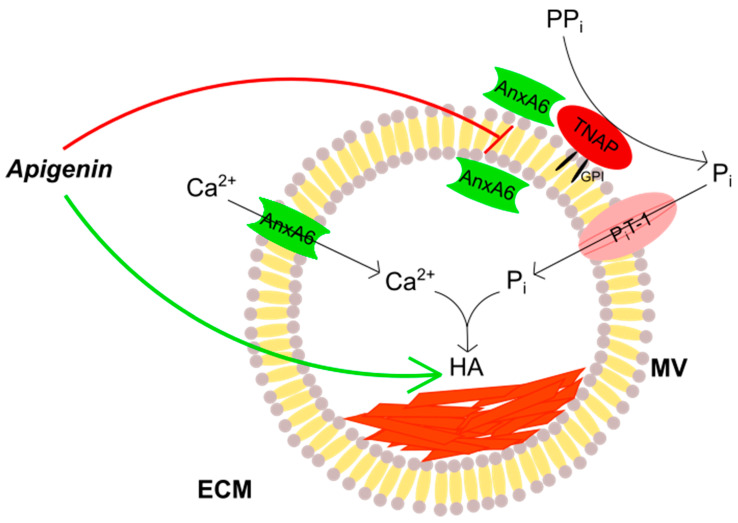
Scheme of the effects of apigenin on the mineralization process. TNAP, attached to the membranes through a glycosylphosphatidylinositol (GPI) anchor, hydrolyzes inorganic pyrophosphate (PP_i_) into inorganic phosphate (P_i_). AnxA6 may function as an inner and outer membrane protein as well as a transmembrane calcium ions (Ca^2+^) channel, whereas P_i_T may function as a P_i_ transporter. Apigenin stimulates (green arrow) hydroxyapatite (HA) formation from Ca^2+^ and P_i_ ions and inhibits (red block) AnxA6 and TNAP co-localization at the membrane of matrix vesicle (MV) secreted by the mineralizing cells to the extracellular matrix (ECM).

To conclude, apigenin has the ability to modulate in vitro the mineralization process carried out by MVs in human bone cells by regulating AnxA6 and TNAP. Our results also confirmed that these proteins play crucial roles in controlling the formation of minerals. What is more, we proved that apigenin has different effects on normal and cancer bone tissues as well as on atherosclerotic soft tissue. It stimulates mineral formation and inhibits AnxA6 and TNAP co-localization at the vesicular membranes (Figure 9). It should be taken into consideration, however, that the dosage of this flavonoid used in our study may never be achievable in real practice, therefore apigenin can be considered as one of the polyphenols that would be good to have present in the diet, but it may not be able to provide full effects observed in vitro.

In summary, the obtained results could help with understanding the mechanisms of apigenin-modulated mineralization and in the development of novel therapies for bone cancer treatment.

## 4. Materials and Methods

### 4.1. Cell Culture and Treatment

Human fetal hFOB 1.19 SV40 large T-antigen transfected osteoblastic cells (ATCC CRL-11372, LGC Standards, Warsaw, Poland) were cultured in a 1:1 mixture of Ham’s F12 medium and Dulbecco’s modified Eagle’s medium with L-glutamine (Sigma-Aldrich, Warsaw, Poland) supplemented with 100 U/mL penicillin, 100 U/mL streptomycin (Sigma-Aldrich, Warsaw, Poland), 0.3 mg/mL G418 (Sigma-Aldrich, Warsaw, Poland), and 10% fetal bovine serum (*v*/*v*, FBS, Gibco, Thermo Fisher Scientific, Warsaw, Poland). The cells were grown at 34 °C in an atmosphere of 5% CO_2_.

Human osteosarcoma Saos-2 cells (ATCC HTB-85, LGC Standards, Warsaw, Poland) were cultured in McCoy’s 5A medium with L-glutamine (Sigma-Aldrich, Warsaw, Poland) supplemented with 100 U/mL penicillin, 100 U/mL streptomycin (Sigma-Aldrich, Warsaw, Poland) and 15% FBS (*v*/*v*, Gibco, Thermo Fisher Scientific, Warsaw, Poland). The cells were grown at 37 °C in an atmosphere of 5% CO_2_.

Human coronary artery smooth muscle cells (HCASMC, C-12511, donor 425Z019.2, PromoCell, Warsaw, Poland) were cultured in Smooth Muscle Cell Growth Medium 2 (PromoCell, Warsaw, Poland) supplemented with a supplement mix containing FCS 0.05 mL/mL, huEGF 0.5 ng/mL, huFGF 2 ng/mL, huInsulin 5 mg/mL (PromoCell, Warsaw, Poland). The cells were grown at 37 °C in the atmosphere of 5% CO2.

Saos-2, hFOB 1.19 and HCASMC cells were stimulated for mineralization at least one day after cell passage and attachment by treatment with 50 µg/mL ascorbic acid (AA, Sigma-Aldrich, Warsaw, Poland) and 7.5 mM β-glycerophosphate (β-GP, Sigma-Aldrich, Warsaw, Poland) for 7 days [57]. The mineralization process was modulated by the addition of different concentrations of apigenin (Extrasyntese, Genay, France) for 1, 3, 5, 7 or 14 days starting 43 h after the addition of AA and β-GP. The final concentration of DMSO, as a solvent for apigenin solutions, in the culture medium did not exceed 0.5% (*v*/*v*). Cell cultures were observed under an inverted Axiovert 40C light microscope (Carl Zeiss, Poznan, Poland) at 100× magnification with Phase contrast.

### 4.2. Morphological Analysis of Cell Culture

Hematoxylin and eosin staining was used for the morphological analysis of cell cultures. Cells were washed with phosphate buffer saline (PBS, pH 7.4), fixed with 3% (*w*/*v*) paraformaldehyde in PBS (20 min, room temperature) [5], followed by hematoxylin and eosin staining using an H&E Stain Kit (Abcam, Cambridge, UK). Cells were washed with deionized water, stained with Hematoxylin, Mayer’s (Lillie’s Modification) for 5 min, washed two times with deionized water for 10 min, incubated with Bluing Reagent for 15 s, washed again two times with deionized water for 10 min, washed with absolute alcohol and dried. Then, cells were stained with Eosin Y Solution (Modified Alcoholic) for 3 min and dehydrated in three changes of absolute alcohol. Stained cell cultures were photographed using an inverted Axiovert 40C light microscope (Carl Zeiss, Poznan, Poland) at 400× magnification with Phase contrast.

### 4.3. MTT Assay

MTT assay was performed in order to determine the effects of apigenin on cell viability of both examined cell lines [50]. Cells were seeded in 96-well plate at a density of 5 × 10^3^ per well and cultured for 24 h prior to treatment with different concentrations of apigenin (1–20 µM). After 1, 3, 5, 7 or 14 days of culture, the cells were washed with PBS and incubated in 0.5 mg/mL 3-(4,5-dimethyl-2-thiazolyl)-2,5-diphenyl-2H-tetrazolium bromide (MTT; Sigma-Aldrich) in medium without serum at 37 °C for 4 h. Then, the precipitated formazan crystals were dissolved by the addition of 40 mM HCl in isopropanol and incubation for 30 min at room temperature. The absorbance was measured at 570 nm using a Spectra Max M5e multi-detection reader (Molecular Devices, San Jose, CA, USA).

### 4.4. Calcium Minerals Detection

The presence of calcium deposits in cells treated with different concentrations of apigenin was detected by staining with Alizarin Red-S (AR-S; Sigma-Aldrich, Warsaw, Poland) [58]. Cell monolayers in 6-well plates (9.6 cm^2^/well) were washed with PBS and incubated with 0.5% (*w*:*v*) AR-S in PBS, pH 5.0, for 30 min at room temperature. Then, cells were washed two times with PBS to remove free calcium ions, and calcium deposits attached to the cells were photographed under an inverted Axiovert 40C light microscope (Carl Zeiss, Poznan, Poland). The quantitative analysis of calcium salts levels in the cells was assessed by de-staining with cetylpyridinium chloride (CPC, Sigma-Aldrich, Warsaw, Poland) [59]. Cells were incubated with 2.5 mL of 10% (*w*:*v*) CPC in PBS, pH 7.0, for 30 min at room temperature. The obtained solution was transferred to centrifuge tubes and centrifuged at 130× *g* for 3 min at room temperature (MPW-350R, MPW Medical Instrument). The collected supernatant was used for measurements of absorbance at 562 nm using a Spectra Max M5e multi-detection reader (Molecular Devices, San Jose, CA, USA). [Ca^2+^] was calculated and referenced to the standard curve of AR-S as described earlier [60]. The degree of mineralization was normalized to the relative number of viable cells as determined using the MTT proliferation assay [36,53].

### 4.5. Cell Lysis and TNAP Acivity Assay

Cells from 5 plates (in a density of 10^8^ per plate), either resting or stimulated and treated with different concentrations of apigenin, were lysed in TLB (1% Triton X-100, 10 mM Na_2_HPO_4_, 1.8 mM KH_2_PO_4_, 2.7 mM KCl, 136 mM NaCl, Protease Inhibitor Cocktail (Sigma-Aldrich, Warsaw, Poland), 0.05 mM PMSF, pH 7.4). Medium from cell cultures was removed, while cells were washed with PBS and incubated with 0.5 mL of TLB at 4 °C. Then, cells were mechanically scraped on ice, passed 10 times through a 0.5 × 16 syringe, and centrifuged at 500× *g* for 5 min at 4 °C (MPW-350R, MPW Medical Instrument, Warsaw, Poland). The collected supernatant was analyzed for TNAP activity using the alkaline phosphatase (ALP) Yellow pNPP (p-nitrophenyl phosphate) Liquid Substrate System for ELISA (Sigma-Aldrich, Warsaw, Poland) as described earlier [11]. The reaction was initiated by the addition of 10 µL (0.5 µg of protein) aliquots of the supernatant to 96-well plates containing 200 µL of pNPP as substrate. The plates were incubated at 37 °C, and the absorbance was measured at 405 nm for 1 h with 15 s intervals using a Spectra Max M5e multi-detection reader (Molecular Devices, San Jose, CA, USA). TNAP activity was calculated as U, where 1 U = 1 µmol pNPP hydrolyzed per min. The TNAP activity values were normalized to the relative number of viable cells as determined using the MTT proliferation assay [36,53].

### 4.6. SDS-PAGE and Immunoblot Analysis

Proteins of cell lysates were separated on 10% (*w*/*v*) SDS-PAGE [61] and then electrotransferred (Mini-Protean*^®^* II^TM^ Kit, Bio-Rad, Hercules, CA, USA) onto nitrocellulose membranes (Hybond^TM^-ECL^TM^, Amersham Biosciences, GE Healthcare, Little Chalfont, UK) according to Towbin et al. [62]. Nitrocellulose membranes were blocked with 5% (*w*/*v*) milk in TBS for 1 h at room temperature. The membranes were then incubated with mouse monoclonal anti-annexin A6 (AnxA6; 1:2500, *v*/*v*; BD Transduction Laboratories, Warsaw, Poland), rabbit polyclonal anti-TNAP (TNAP; 1:250, *v*/*v*; Abcam, Cambridge, UK), or mouse monoclonal anti-beta-actin (Actin; 1:40,000, *v*/*v*; Sigma, Warsaw, Poland) primary antibodies prepared in 5% (*w*/*v*) milk in TBS supplemented with 0.05% (*v*/*v*) Tween-20 (TBST), at 4 °C overnight. Nitrocellulose membranes were washed several times with TBST and then incubated for 2 h at room temperature with sheep anti-mouse or anti-rabbit IgG secondary antibodies conjugated with horseradish peroxidase (1:5000, *v*/*v*; Cell Signaling Technology, Danvers, MA, USA) and prepared in 5% (*w*/*v*) milk in TBST. Finally, the membranes were washed, and immunoreactive bands were visualized on MXB X-ray films (Kodak) using ECL reagents according to the manufacturer’s instructions (Millipore, Burlington, MA, USA). Then the films were analyzed densitometricaly using the InGenius software (Syngene, Cambridge, UK).

### 4.7. Immunochemistry and Fluorescent Microscopy

A total of 10^5^ cells were cultured in culture medium on coverslips overnight at 37 °C in 5% CO_2_ humidified atmosphere. The next day, the stimulators (50 µg/mL AA and 7.5 mM β-GP) and, 3 h later, apigenin at different concentrations, were added to the appropriate cell culture variants for 7 days. Then cells were washed with PBS and fixed with 3% (*w*/*v*) paraformaldehyde in PBS (20 min, room temperature) [5]. After washing with PBS, fixed cells were incubated in 50 mM NH_4_Cl in PBS (10 min, room temperature), washed with PBS, and then permeabilized with 0.08% (*v*/*v*) Triton X-100 in PBS (5 min, 4 °C). After additional washing with Tris-buffered saline (TBS; 100 mM NaCl, 10 mM Tris-HCl, and pH 7.5), cells were incubated in a blocking solution, 5% (*v*/*v*) FBS in TBS (60 min, room temperature). Then, cells were incubated with mouse monoclonal anti-annexin A6 (AnxA6; 1:100, *v*/*v*; BD Transduction Laboratories, Warsaw, Poland) and rabbit polyclonal anti-TNAP (TNAP; 1:50, *v*/*v*; Abcam, Cambridge, UK) primary antibodies diluted in TBS containing 0.5% FBS (*v*/*v*) and 0.05% Tween-20 (*v*/*v*) (overnight, 4 C). Then, the cells were washed 5 times with TBS containing 0.5% FBS (*v*/*v*) and 0.05% Tween-20 (*v*/*v*) and incubated for 1 h at room temperature with chicken anti-mouse IgG Alexa Fluor 488 (1:400, *v*/*v*; Invitrogen, Waltham, MA, USA) and chicken anti-rabbit IgG Alexa Fluor 594 (1:200, *v*/*v*; Invitrogen, Waltham, MA, USA) secondary antibodies diluted in TBS containing 0.5% FBS (*v*/*v*) and 0.05% Tween-20 (*v*/*v*). After washing several times with TBS containing 0.5% FBS (*v*/*v*) and 0.05% Tween-20 (*v*/*v*), once in TBS and deionized water, cover slips were mounted with ProLong^®^ Gold Antifade Reagent (Cell Signaling Technology, Danvers, MA, USA) on microscope slides. Samples were stored at room temperature overnight and then photographed under an AxioObserver.Z1 fluorescent microscope (Carl Zeiss, Poznan, Poland) at 630× magnification with appropriate fluorescent filters.

The co-localization coefficients were measured by AxioVision Rel. 4.8 software (Carl Zeiss, Poznan, Poland) in FM images for each channel as described earlier [13].

### 4.8. Transmission Electron Microscopy with X-ray Microanalysis (TEM-EDX)

Cells, either resting or stimulated and treated with different concentrations of apigenin for 7 days, were lysed in TLB in the same way as described in Section 4.5. Then, 10 µL of the obtained pellets were negatively stained as follows: the pellets were placed on Formvar/Carbon 300 mesh Cu grids (Agar Scientific, Stansted, UK), allowed to rest at room temperature for 30 min, stained with 2.5% uranyl acetate solution in 50% ethanol in darkness at room temperature for 15 min, washed once in 50% ethanol and three times in deionized water and dried at room temperature for 24 h. The prepared samples were observed under a JEM 1400-TEM (Jeol Co., Tokyo, Japan) transmission electron microscope equipped with an INCA energy dispersive X-ray microanalysis (EDX) system (Oxford Instruments, Oxfordshire, UK) and an 11 Mega pixel MORADA G2 camera (Olympus Soft Imaging Solutions, Tokyo, Japan). Images were taken at a magnification of 50,000×. Mapping of Ca, P and Cl distribution was performed by INCA Suite version 4.11 software, System No. 46321 (Oxford Instruments, Oxfordshire, UK).

### 4.9. Transmission Electron Microscopy with Immunogold Labelling (TEM-gold)

Cells, either resting or stimulated and treated with different concentrations of apigenin for 7 days, were lysed in TLB in the same way as described in Section 4.5. Then, 10 µL of the obtained pellets were negatively stained as follows: they were placed on Formvar/Carbon 300 mesh Ni grids (Agar Scientific, Stansted, UK), incubated at room temperature for 30 min, stained with 2.5% uranyl acetate solution in 50% ethanol in darkness at room temperature for 10 min, washed once in 50% ethanol and three times in deionized water, and dried at room temperature for 24 h. Then samples were blocked with 5% (*v*/*v*) FBS in PBS (60 min, room temperature), washed with PBS, and incubated with mouse monoclonal anti-annexin A6 (AnxA6; 1:5, *v*/*v*; BD Transduction Laboratories, Warsaw, Poland) and rabbit polyclonal anti-TNAP (TNAP; 1:5, *v*/*v*; Abcam, Cambridge, UK) primary antibodies diluted in PBS containing 0.5% FBS (*v*/*v*) and 0.05% Tween-20 (*v*/*v*) (overnight, room temperature). After that, samples were washed 6 times with PBS containing 0.5% FBS (*v*/*v*) and 0.05% Tween-20 (*v*/*v*) and incubated for 3.5 h at room temperature with 10 nm colloidal gold-conjugated goat anti-mouse IgG (1:20, *v*/*v*; Sigma-Aldrich, Warsaw, Poland) and 5 nm colloidal gold-conjugated goat anti-rabbit IgG (1:20, *v*/*v*; Sigma-Aldrich, Warsaw, Poland) secondary antibodies diluted in PBS containing 0.5% FBS (*v*/*v*) and 0.05% Tween-20 (*v*/*v*). After washing 6 times with PBS containing 0.5% FBS (*v*/*v*) and 0.05% Tween-20 (*v*/*v*), 2 times with PBS and once with deionized water, grids were stained with 2.5% uranyl acetate solution in 50% ethanol in darkness at room temperature for 20 min, washed once in 50% ethanol and three times in deionized water and dried at room temperature for 24 h. The prepared samples were observed under a JEM 1400-TEM (Jeol Co., Tokyo, Japan) transmission electron microscope equipped with an 11 Mega pixel MORADA G2 camera (Olympus Soft Imaging Solutions, Tokyo, Japan). Images were taken at a magnification of 100,000× and 300,000×.

### 4.10. Statistical Analysis

All the values are reported as mean ± SD. Data were analyzed by one-way ANOVA, and post hoc analyses were performed using the Tukey method with the aid of Daniel’s XL Toolbox add-in for Excel, version 6.60, by Daniel Kraus, Wurzburg, Germany [63]. Statistical significance was described as * *p* < 0.05, ** *p* < 0.01, and *** *p* < 0.001.

## Figures and Tables

**Table 1:** Relative co-localization area calculated for AnxA6 vs. TNAP in hFOB 1.19 and Saos-2 cells under resting conditions or in the presence of stimulators, AA and β-GP. Cells were incubated with different concentrations (µM) of apigenin for 7 days. Data are the means ± S.E. of at least three independent experiments (* *p* < 0.05, ** *p* < 0.01).

	Apigenin Concentration (µM)	Relative Co-Localization Area (%)			
		hFOB 1.19		Saos-2	
**Resting**	0	11.34 ± 3.00	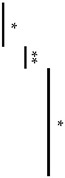	15.40 ± 3.93	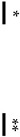
	1	19.90 ± 4.23		8.48 ± 1.14	
	2	19.40 ± 2.04		7.88 ± 0.67	
	5	12.53 ± 0.76		6.28 ± 2.79	
	10	10.34 ± 3.53		4.59 ± 0.20	
**Stimulated**	0	13.18 ± 5.92		17.93 ± 2.33	
	1	12.86 ± 5.92		8.40 ± 1.07	
	2	13.96 ± 3.84		6.50 ± 0.69	
	5	8.09 ± 1.77		6.11 ± 2.23	
	10	8.53 ± 3.48		5.59 ± 1.94	

## Data Availability

Not applicable.

## Supplementary Materials

The following are available online at https://www.mdpi.com/article/10.3390/ijms232113179/s1.

## Abbreviations

AA	ascorbic acid
ALP	alkaline phosphatase
AnxA6	vertebrate annexin A6
AR-S	Alizarin Red-S
β-GP	β-glycerophosphate
CPC	cetyl pyridinium chloride
ECM	extracellular matrix
FBS	fetal bovine serum
HA	hydroxyapatite
MVs	matrix vesicles
PBS	phosphate-buffered saline
P_i_	inorganic phosphate
pNPP	para-nitro phenyl phosphate
PP_i_	inorganic pyrophosphate
SMC	smooth muscle cells
TBS	Tris-buffered saline
TNAP	tissue non-specific alkaline phosphatase
