# Supplementary material for: Apigenin Modulates AnxA6- and TNAP-Mediated Osteoblast Mineralization
Source: Int J Mol Sci. 2022 Oct 29;23(21):13179. doi: 10.3390/ijms232113179 (PMC9658728; doi:10.3390/ijms232113179)
Supplement: Supplementary file 1 [file ijms-23-13179-s001.zip › ijms-1995590-supplementary.pdf]

Table S1. The effect of apigenin on viability of hFOB 1.19 (A), Saos-2 (B) and HCASMC (C) cells. The cells were incubated with different concentrations (μM) of apigenin for 1, 3, 5, 7, 14 days (hFOB 1.19 and Saos-2 cells) or 2, 4, 7, 14, 21 days (HCASMC cells), and then MTT assay was performed. Data are means ± S.E. of at least three independent experiments (* p<0,05, ** p<0,01 compared with cells without apigenin).

A

hFOB 1.19

**Apigenin concentration (μM)**	**Absorbance**				
	**1 day**	**3 days**	**5 days**	**7 days**	**14 days**
0	0.136 ± 0.014	0.161 ± 0.034	0.224 ± 0.019	0.273 ± 0.051	0.385 ± 0.030
1	0.132 ± 0.017	0.151 ± 0.006	0.212 ± 0.013	0.261 ± 0.030	0.432 ± 0.024
2	0.137 ± 0.023	0.158 ± 0.013	0.190 ± 0.021	0.216 ± 0.029	0.351 ± 0.036
5	0.132 ± 0.028	0.148 ± 0.030	0.175 ± 0.008	0.204 ± 0.043	0.236 ± 0.036
10	0.119 ± 0.018	0.128 ± 0.010	0.135 ± 0.007	0.174 ± 0.043	0.130 ± 0.024
20	0.121 ± 0.027	0.113 ± 0.017	0.115 ± 0.017	0.121 ± 0.029	0.113 ± 0.017

B

Saos-2

**Apigenin concentration (μM)**	**Absorbance**				
	**1 day**	**3 days**	**5 days**	**7 days**	**14 days**
0	0.102 ± 0.015	0.162 ± 0.030	0.266 ± 0.008	0.340 ± 0.024	0.620 ± 0.073
1	0.117 ± 0.018	0.185 ± 0.028	0.251 ± 0.035	0.272 ± 0.031	0.504 ± 0.061
2	0.109 ± 0.010	0.179 ± 0.021	0.230 ± 0.037	0.245 ± 0.030	0.391 ± 0.022
5	0.113 ± 0.027	0.151 ± 0.030	0.203 ± 0.015	0.229 ± 0.028	0.389 ± 0.016
10	0.116 ± 0.015	0.118 ± 0.022	0.162 ± 0.024	0.191 ± 0.022	0.269 ± 0.046
20	0.094 ± 0.012	0.094 ± 0.016	0.088 ± 0.011	0.092 ± 0.014	0.082 ± 0.012

C

HCASMC

**Apigenin concentration (μM)**	**Absorbance**				
	**2 day**	**4 days**	**7 days**	**14 days**	**21 days**
0	0.133 ± 0.019	0.201 ± 0.098	0.035 ± 0.015	0.145 ± 0.042	0.110 ± 0.048
1	0.245 ± 0.017	0.047 ± 0.027	0.081 ± 0.008	0.145 ± 0.002	0.251 ± 0.071
2	0.234 ± 0.075	0.066 ± 0.041	0.100 ± 0.020	0.115 ± 0.007	0.382 ± 0.036
5	0.149 ± 0.015	0.071 ± 0.041	0.103 ± 0.026	0.051 ± 0.042	0.385 ± 0.035
10	0.173 ± 0.056	0.125 ± 0.095	0.140 ± 0.014	0.030 ± 0.046	0.365 ± 0.028
20	0.292 ± 0.024	0.208 ± 0.081	0.112 ± 0.012	0.099 ± 0.020	0.203 ± 0.244

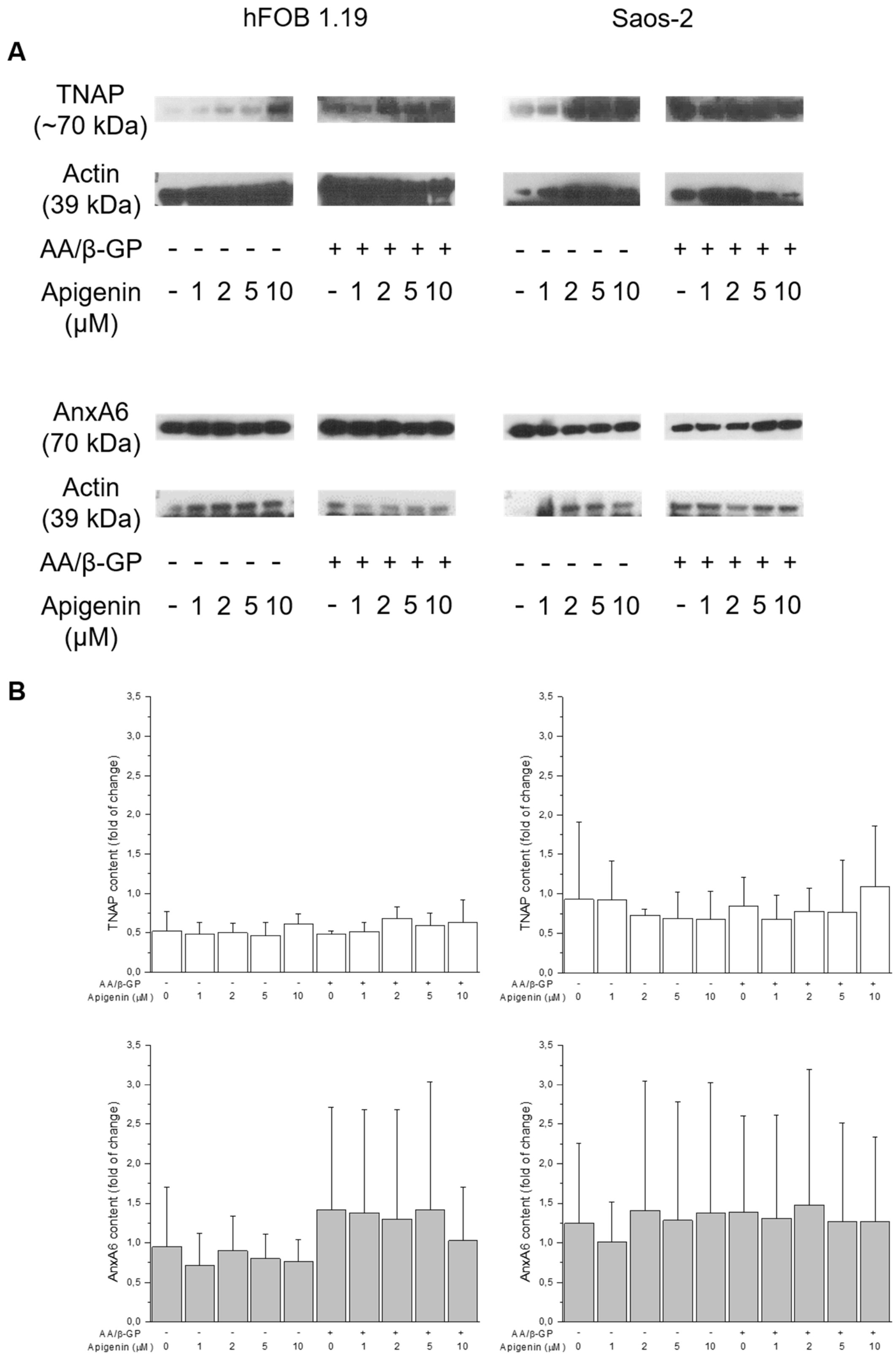

Figure S1. Protein level determined by Western Blot (WB) of TNAP, AnxA6 and actin in hFOB 1.19 and Saos-2 cells under resting conditions (R) or after 7 days stimulation with AA and β-GP (S) in control cells and cells subjected to treatment with different concentrations (μM) of apigenin. Whole cell lysates were prepared in TLB. **(A)** WB were incubated with rabbit anti-TNAP and mouse anti-AnxA6 primary antibodies followed by appropriate secondary antibodies conjugated with horseradish peroxidase. The proteins content was quantified using inGenius software (Syngene) and calculated per actin content. **(B)** Data are means ± S.E. of at least three independent experiments.

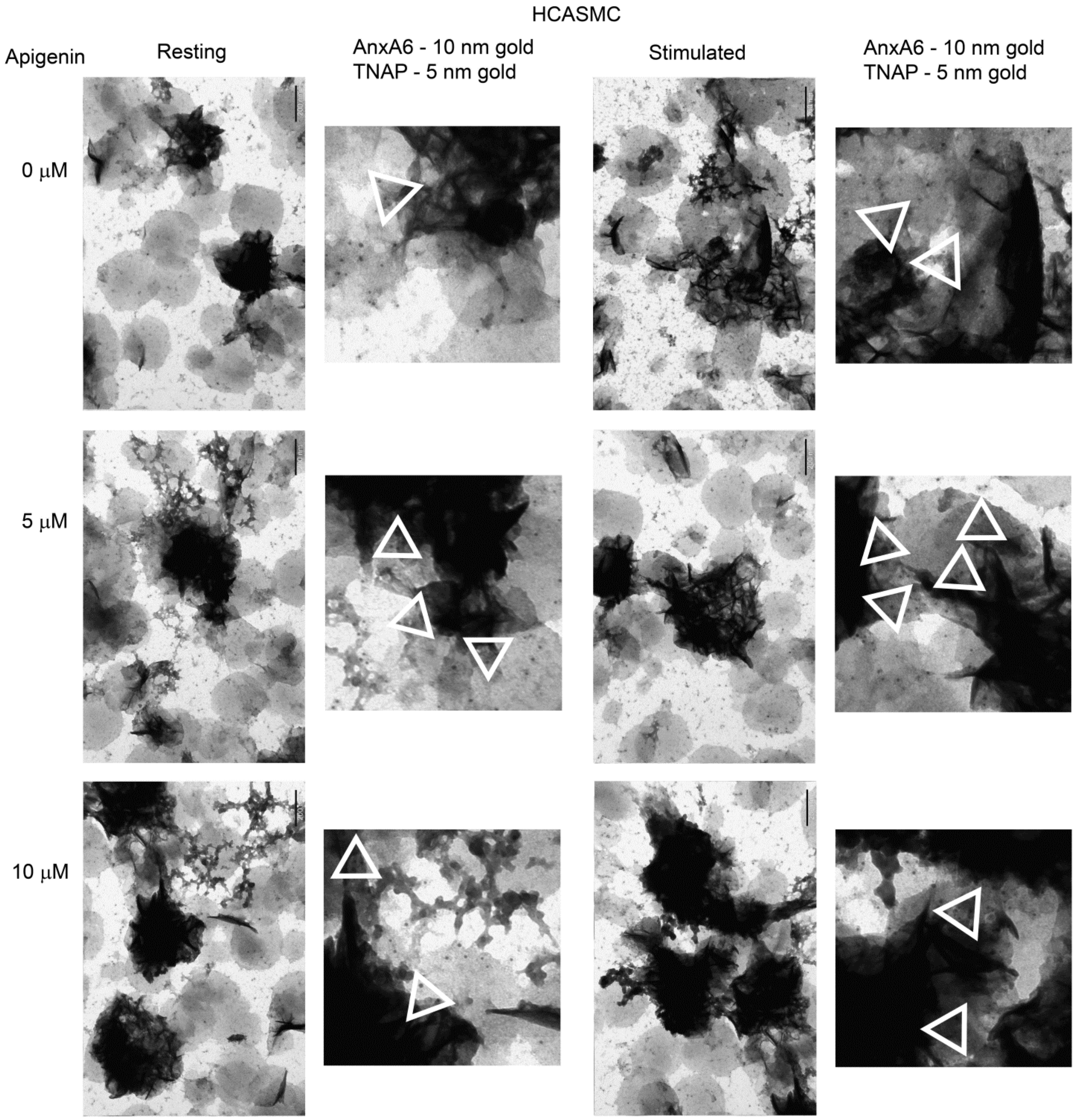

Figure S2. TEM images of co-localization of AnxA6 and TNAP in MVs during mineralization of HCASMC cells under resting conditions or in the presence of stimulators, AA and β-GP. Cells were incubated with different concentrations (μM) of apigenin for 14 days, lysed and analyzed by TEM (magnification 100,000×). Bar: 200 nm. Additional magnifications 300,000×. AnxA6 was labelled with anti-AnxA6 primary antibody conjugated with 10 nm colloidal gold secondary antibody. TNAP was labelled with anti-TNAP primary antibody conjugated with 5 nm colloidal gold secondary antibody. Sites of AnxA6 and TNAP co-localization are marked by triangles for HCASMC cells.
